# Double Trouble: Synchronous Hepatoduodenal and Hepatocolic Fistulisation in a Case of Untreated Liver Abscess

**DOI:** 10.7759/cureus.63633

**Published:** 2024-07-01

**Authors:** Saket Dadhich, Vijay Vatvani, Hitakshi Chahal, Richa Jain, Hanuman R Khoja

**Affiliations:** 1 General Surgery, Sawai Man Singh Medical College, Jaipur, IND; 2 Radiology, Sawai Man Singh Medical College, Jaipur, IND

**Keywords:** air pocket, rupture, hepatic abscess, hepatocolic fistula, hepatoduodenal fistula

## Abstract

A hepatic abscess is a potentially life-threatening condition that can lead to major complications if left untreated. One of the rarest complications of hepatic abscesses is rupture into adjacent organs like the duodenum, stomach, and colon. We report a case of an elderly male patient with a hepatic abscess that ruptured into the duodenum and colon, forming fistulous connections. To the best of our knowledge, this is the only reported case of hepatic abscess with simultaneous hepatoduodenal and hepatotoxic fistulas, confirmed radiologically and endoscopically. This report signifies the need for a high level of vigilance for extremely rare complications in relatively common conditions.

## Introduction

A hepatic abscess, also known as a liver abscess, is a collection of pus in the liver as a result of infection. Liver abscesses are the most common type of visceral abscess [1]. Overall, these conditions are rare, with more cases reported in developing nations. India has the second-highest incidence of liver abscess in the world [2]. This condition can lead to serious morbidity and even mortality in the absence of prompt attention [3]. Due to the subtle and chronic presentation in the form of abdominal pain, intermittent fever, nausea, vomiting, anorexia, and sometimes jaundice, such cases are often delayed in diagnosis and management. Although extremely rare, enterohepatic fistulas may result from the rupture of an untreated liver abscess. This is the first diagnostically confirmed report of a simultaneous incidence of hepatoduodenal and hepatocolic fistulas in an untreated liver abscess. This report highlights the need for a high index of suspicion for extremely rare complications in a condition that may be relatively common, especially in countries with poor sanitation.

## Case presentation

A 69-year-old chronic alcoholic male presented with a history of dull, aching right upper abdominal pain with mild to moderate intensity, which was insidious in onset, with no radiation, associated with a low-grade fever for one month. The patient also had complaints of five episodes of vomiting of reddish-brown material two days before admission, after which the pain was partially relieved. There was no history of yellowish discoloration of the skin, dark-coloured stools, any drug use, or any previous hospitalisation/instrumentation. He was a smoker and an alcoholic for 30 years. There were no other associated comorbidities. On examination, the patient had stable vitals and was mildly febrile; pallor was present with no jaundice. There was tenderness in the right upper quadrant, with the liver being palpable 5 cm below the subcostal margin. The spleen was not palpable, and no free fluid was noted.

**Figure 1:**
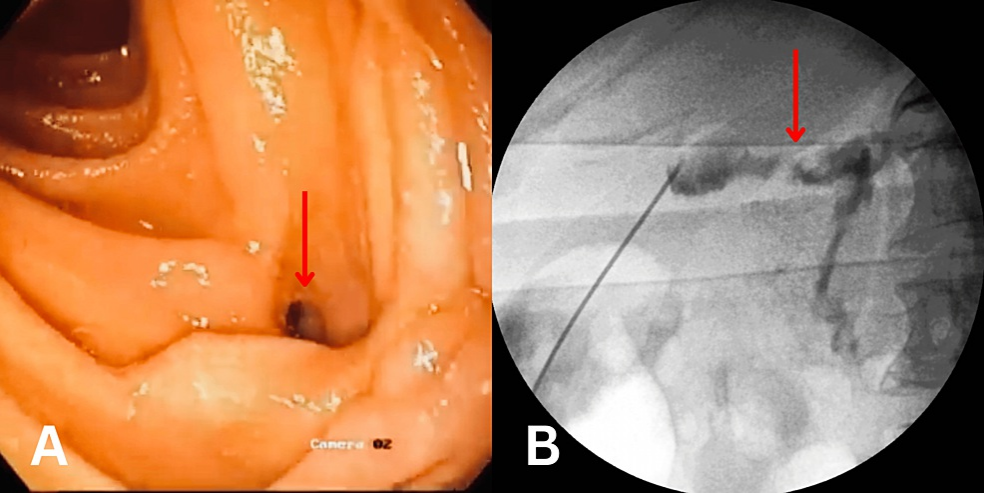
Gastroduodenoscopy and fluoroscopy images (A) Gastroduodenoscopy image showing a fistulous opening (arrow mark) of about 7 mm in the proximal part of the duodenum. (B) Fluoroscopy image showing spillage of instilled dye, establishing the presence of an abnormal connection (arrow mark) between one of the hepatic abscesses and the colon.

**Figure 2:**
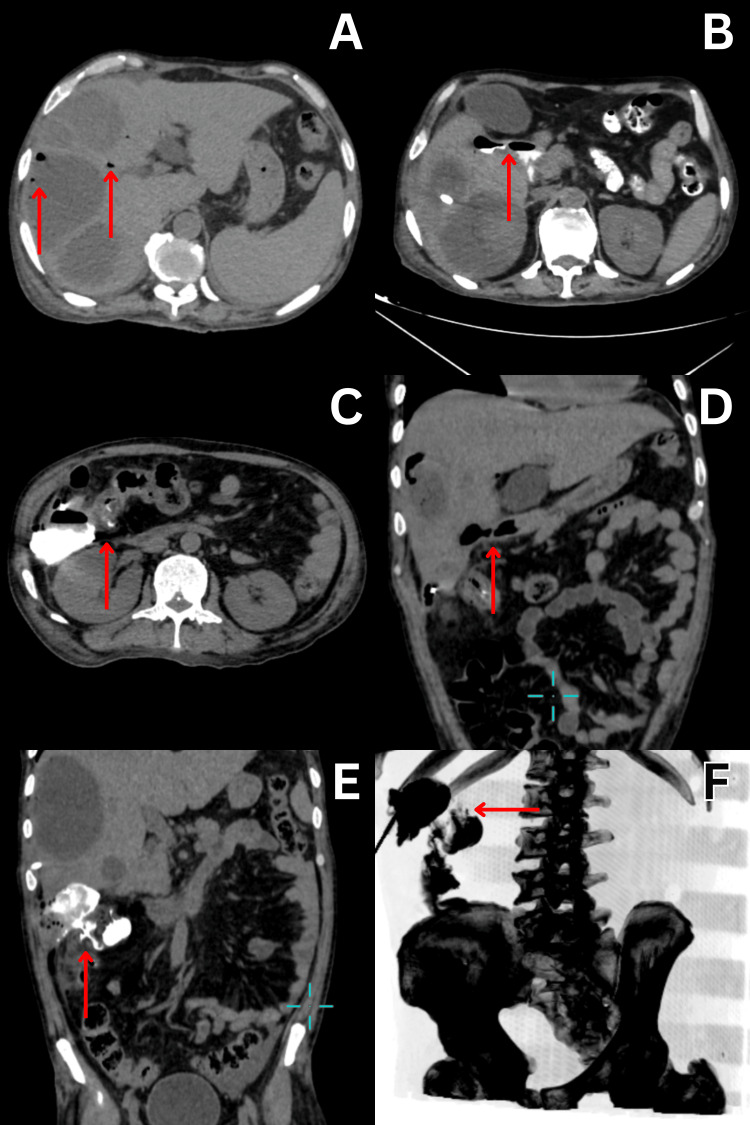
CT abdomen and fluoroscopy images (A) CT abdomen axial section showing multiple hepatic abscesses involving the right lobe of the liver with the presence of multiple air foci (arrow marks). (B) CT abdomen axial section showing abnormal fistulous communication (arrow mark) between the hepatic parenchyma and proximal part of the duodenum with contrast spillage. (C) CT abdomen axial section showing abnormal fistulous communication (arrow mark) between hepatic parenchyma and colon near hepatic flexure with contrast spillage. (D) CT abdomen coronal section showing the presence of an air-filled hepatoduodenal fistula (arrow mark) near the inferior surface of the liver. (E) CT abdomen coronal section showing the presence of a contrast-enhanced hepatocolic fistula (arrow mark) near the hepatic flexure. (F) CT abdomen 3D MIP B/W inverse image showing spillage of instilled contrast (arrow mark) through the hepatocolic fistula.

Blood investigations revealed anaemia with haemoglobin 5.8 g/dl and a total leukocyte count of 18,400/cc with 83.2% neutrophils. The erythrocyte sedimentation rate was 83 mm/hour. Serum bilirubin was 1.57 mg/dl, aspartate transaminase and alanine transaminase were 73 IU/L and 29 IU/L, and alkaline phosphatase was 337 IU/L. Ultrasonography of the abdomen revealed hepatomegaly with cystic lesions of variable sizes in both lobes of the liver, some containing air foci. Gastroduodenoscopy was performed in view of reddish-brown vomiting, which demonstrated a fistulous opening of diameter 7 mm in the proximal part of the duodenum (Figure 1A); colonoscopy could not demonstrate any abnormal findings due to inadequate preparation. CT abdomen and fluoroscopy were performed to identify the origin of intrahepatic air foci. Multiple heterogenous lesions were seen in both lobes of the liver with multiple air foci, the largest collection being of size 49 mm × 46 mm × 42 mm in the right lobe (Figure 2A). The presence of an abnormal fistulous connection between one of the cavities and the hepatic flexure was confirmed after the instillation of contrast into the cavity percutaneously (Figure 1B). Two air containing abnormal communications with oral contrast spillage were observed in the hepatic parenchyma near the proximal part of the duodenum and hepatic flexure (Figure 2B-2F).

The patient did not consent to surgical management. Hence, it was managed with percutaneous drainage of large collections along with intravenous metronidazole and symptomatic management according to the institute protocol. Around 1.5 L of yellowish-brown, foul-smelling pus was drained from the catheter over five days, after which repeat ultrasonography was performed to demonstrate minimal fluid presence in the cavity, and the catheter was removed. The pus culture grew two organisms, namely Escherichia coli and Klebsiella pneumonia, and the antibiotic therapy was modified as per the culture report. The patient was discharged with no additional complaints and an improved general condition. Eight-week follow-up gastroduodenoscopy showed a decreased diameter of the duodenal fistulous opening to 4 mm.

## Discussion

Liver abscess is an endemic form of intra-abdominal infection in India. Its incidence is on the rise with changing food and alcohol consumption habits. They are broadly divided into amoebic type (caused by Entamoeba hystolitica) and pyogenic type, which is further classified on the basis of bacterial and fungal aetiology. Hepatic abscesses present variably in terms of clinical symptoms and radiological imaging [4]. Patients usually complain of epigastric or right upper quadrant (RUQ) pain, low-grade intermittent fever, anorexia with elicitable tenderness over RUQ, hepatomegaly, or palpable mass, along with jaundice and ascites. They are common in men of older age with comorbidities such as diabetes, malignancy, previous biliary surgery, or an immunosuppressive state [5]. These cases are also commonly found in areas with poor sanitation, water logging, overcrowding, and malnourishment.

Ultrasonography, along with blood investigations, is the first step in establishing the diagnosis and extent of this disease. Amoebic abscess commonly presents as an isolated lesion with a predisposition to the right lobe of the liver. On the other hand, pyogenic varieties are seen as multiple collections that may be present in any segment of the liver [6]. The presence of air foci on ultrasonography of a hepatic abscess should raise the suspicion of a hepatobronchial fistula, a hepatoenteric fistula, or a secondary bacterial infection [7]. Rupture of a hepatic abscess into the peritoneal or pleural cavity is relatively common [8], while the formation of enterohepatic fistulas is an extremely rare phenomenon seen in less than 1.5% of cases [9]. Instillation of contrast in the abscess cavity, a water-soluble barium swallow, or CT with oral contrast may be used to confirm the diagnosis of a hepatoenteric fistula [10]. In cases of untreated liver abscess, common sites of rupture and fistula formation include the stomach, colon, and duodenum [7,9,10]. The endoscopic assessment also becomes of critical value in such cases due to the direct communication of the liver with the gastrointestinal system, which may lead the patient into ascending infections, cholangitis, and other complications [11].

Since it was first described in 1983, only 16 cases have been published reporting rupture of a liver abscess with fistulisation into the gastrointestinal tract as sequelae [12]. This establishes enterohepatic fistulas as an extremely rare complication of liver abscess. Without any clear consensus regarding the management of such complications, surgery is considered to be the mainstay of treatment for the closure of the enterohepatic fistula. However, reports advocating the use of a conservative approach and antibiotic therapy have also been published [13]. A delay in the percutaneous drainage of a liver abscess has also been implicated in favour of such complications [14]. In the literature to date, no case has been reported with the simultaneous presence of both hepatoduodenal and hepatocolic fistulas in an untreated patient with a liver abscess without any history of intervention.

## Conclusions

The development of both hepatoduodenal and hepatocolic fistulas simultaneously is an extremely rare finding. These complications may provide a serious increment in the morbidity of an otherwise manageable disease. A high degree of suspicion is warranted for early diagnosis using all radiological and endoscopic modalities at disposal. In the absence of any established treatment guidelines, focus should be given to stop further worsening of the condition and steady recovery. High-quality imaging modalities and the availability of image-guided percutaneous drainage have significantly lowered mortality and increased the standard of patient care. Further research and technological advancements may refine the management protocol.

## References

[1] Altemeier WA, Culbertson WR, Fullen WD, Shook CD (1973). Intra-abdominal abscesses. Am J Surg.

[2] Fazul Ur, Choudhari RB, Patil AG (2014). A clinical study, diagnosis and management of liver abscess at VIMS, Bellary. Jebmh.

[3] Adams EB, MacLeod IN (1977). Invasive amebiasis. II. Amebic liver abscess and its complications. Medicine (Baltimore).

[4] Halvorsen RA, Korobkin M, Foster WL, Silverman PM, Thompson WM (1984). The variable CT appearance of hepatic abscesses. AJR Am J Roentgenol.

[5] Gupta M, Sharma A, Singh R, Lehl SS (2013). Citrobacter koseri: an unusual cause of pyogenic liver abscess. BMJ Case Rep.

[6] Lodhi S, Sarwari AR, Muzammil M, Salam A, Smego RA (2004). Features distinguishing amoebic from pyogenic liver abscess: a review of 577 adult cases. Trop Med Int Health.

[7] Lamba AS, Singh B, Gupta M, Dahiya S, Saini R (2020). Hepato-duodenal fistula complicating a pyogenic liver abscess: an unusual presentation. Cureus.

[8] Budhiraja S, Dhatt GS, Babra RS (2006). Hepatogastric fistula in a pediatric patient. Pediatr Surg Int.

[9] Yang DM, Kim HN, Kang JH, Seo TS, Park CH, Kim HS (2004). Complications of pyogenic hepatic abscess: computed tomography and clinical features. J Comput Assist Tomogr.

[10] Mowji PJ, Cohen AJ, Potkin B, Viltuznik J (1987). Amebic liver abscess with hepatoduodenal fistula. Am J Gastroenterol.

[11] Yamada T, Murakami K, Tsuchida K (2000). Ascending cholangitis as a cause of pyogenic liver abscesses complicated by a gastric submucosal abscess and fistula. J Clin Gastroenterol.

[12] Timbol AB, Mondragon KA, Banez VP (2017). Hepatocolic fistula: a rare presentation of pyogenic liver abscess. BMJ Case Rep.

[13] Ishwar M, Tambat R, Srinivas NM (2015). Nature heals: spontaneous resolution of liver abscess through hepato-colic fistula. IJSS Case Rep Rev.

[14] Lee KW, Kim HY, Kim CW (2017). Hepatogastric fistula as a rare complication of pyogenic liver abscess. Clin Mol Hepatol.

